# Rice seed vigor detection based on near-infrared hyperspectral imaging and deep transfer learning

**DOI:** 10.3389/fpls.2023.1283921

**Published:** 2023-10-23

**Authors:** Hengnian Qi, Zihong Huang, Zeyu Sun, Qizhe Tang, Guangwu Zhao, Xuhua Zhu, Chu Zhang

**Affiliations:** ^1^ School of Information Engineering, Huzhou University, Huzhou, China; ^2^ College of Advanced Agricultural Sciences, Zhejiang A&F University, Lin’an, China; ^3^ Smart Agriculture Research Institute, Zhejiang Top Cloud-agri Technology Co., Ltd., Hangzhou, China

**Keywords:** near-infrared hyperspectral imaging, seed vigor, convolutional neural network, fine-tuning, MixStyle

## Abstract

Vigor is one of the important factors that affects rice yield and quality. Rapid and accurate detection of rice seed vigor is of great importance for rice production. In this study, near-infrared hyperspectral imaging technique and transfer learning were combined to detect rice seed vigor. Four varieties of artificial-aged rice seeds (Yongyou12, Yongyou1540, Suxiangjing100, and Longjingyou1212) were studied. Different convolutional neural network (CNN) models were built to detect the vigor of the rice seeds. Two transfer strategies, fine-tuning and MixStyle, were used to transfer knowledge among different rice varieties for vigor detection. The experimental results showed that the convolutional neural network model of Yongyou12 classified the vigor of Yongyou1540, Suxiangjing100, and Longjingyou1212 through MixStyle transfer knowledge, and the accuracy reached 90.00%, 80.33%, and 85.00% in validation sets, respectively, which was better or close to the initial modeling performances of each variety. MixStyle statistics are based on probabilistic mixed instance-level features of cross-source domain training samples. When training instances, new domains can be synthesized, which increases the domain diversity of the source domain, thereby improving the generalization ability of the trained model. This study would help rapid and accurate detection of a large varieties of crop seeds.

## 1 Introduction

Rice is one of the most important crops in the world, serving as the main food crop in many countries around the world and acting as a fundamental food source for mankind (Jin et al., 2022a). Seed vigor is a crucial indicator of seeds, directly affecting their yield (Song et al., 2018). Studying seed vigor before seed germination can help identify high-vigor seeds with higher germination rates and faster growth potential, ultimately improving seed utilization and yield (Du et al., 2017; Zhang J. et al., 2020). Additionally, seed vigor also plays an essential role in evaluating the strengths and weaknesses of different varieties. By studying the seed vigor of various varieties, it is possible to identify excellent varieties that display good vigor. This provides a theoretical foundation for selecting and breeding high-quality rice varieties (Wu et al., 2021), which is conducive to the continuation of excellent varieties. Therefore, it is important to detect the vigor of rice seeds. However, the traditional germination experiment to detect seed vigor is time-consuming and laborious, and other detection methods, such as staining (Carvalho et al., 2017) and electronic conductivity test (Barbosa et al., 2021), will more or less damage and damage seeds, rely on chemical reagents, complex operation, affect seed reuse, at the same time, these detection methods are inefficient, unable to detect rice seed vigor efficiently and non-destructively.

Near-infrared hyperspectral imaging technology is a light-based nondestructive testing technology. It can obtain the spectral information of the object by analyzing the near-infrared energy spectrum reflected or transmitted by the object, so as to achieve rapid quantitative detection of the corresponding parameters of the object (Zhang and Guo, 2019). The specific principle is to use the strong penetration characteristics of near-infrared light, near-infrared light irradiates through the surface of the sample, and the chemical composition, tissue structure and morphology of the sample will have an impact on the absorption, scattering, reflection and other aspects of light (Jin et al., 2022a; Jin et al., 2022b). Near-infrared hyperspectral imaging systems can capture these reactions and present the data in the form of a 3D cube that integrates spectral and spatial information to obtain high-precision imaging results (Zhang L. et al., 2020). In recent years, some scholars have applied NIR-HSI to the detection of rice seed vigor: Jin et al. (2022a) combined NIR-HIS with machine learning and deep learning to predict the seed vigor of different varieties of rice, and the accuracy of most models was more than 85%, in addition, they (Jin et al., 2022b) also used NIR-HSI with LeNet, GoogLeNet, and Residual network (ResNet) to identify rice seed varieties. Among them, the classification effect of the ResNet model was the best. The classification accuracy rate of the test set was 86.08%. In addition, some scholars have also applied NIR-HIS to the detection of rice seed vigor, He et al. (2019) utilized Savitzky-Golay preprocessed extreme learning machine model to detect seed viability in 3 different years, using only 8 bands of spectral data, the classification accuracy was as high as 93.67%. Hong et al. (2022) used models such as partial least squares (PLS) discriminant analysis, support vector machine (SVM), PLS-SVM, PLS-artificial neural network, and one-dimensional convolutional neural network (CNN) to predict vigor using averaging and hyperspectral images. The results show that in most models, about 90% accuracy and a high F1 score can be obtained. The experimental results have proved that the method is efficient, accurate and feasible. However, none of the aforementioned studies addressed the generalization ability of the models, and the models established in these studies were trained from scratch, specifically designed for particular learning tasks.

Deep learning (DL) is an important artificial intelligence method that enables machines to autonomously acquire knowledge from data (Jordan and Mitchell, 2015), and DL is gradually being applied to the field of spectral analysis (Jin et al., 2018; He et al., 2019). In practice, it is not recommended to train neural networks from scratch because it is time-consuming and the performance of the model is not guaranteed. In addition, DL often requires a large amount of data to participate in the training process, and when the amount of data in building the model is not enough, the model may overfit or fall into local optimum (Pan and Yang, 2010; Marmanis et al., 2016; Suh et al., 2018; Lu et al., 2019). Unfortunately, some seed samples are challenging to obtain during germination, leading to high labeling costs and a difficulty in creating large-scale, high-quality datasets. Additionally, data acquired at significant cost is hard to reuse in new tasks, posing challenges for DL, which relies on numerous labeled samples for effective training (Zhang and Zhao, 2021). Transfer learning mainly studies the transfer of knowledge from the source domain to the target domain, allowing the training data and test data to be located in different feature spaces, which has a unique advantage in accelerating the training of the model and improving the generalization performance of the model, with which it can solve the learning problem in the case of small samples, low resources, and few labeled samples (Wu et al., 2021). Transfer learning has been used for the identification of agricultural varieties: Wu et al. (2021) used deep transfer to transfer knowledge to four datasets of rice, oats, wheat, and cotton, and Accuracies of the deep transferred model achieved 95, 99, 80.8, and 83.86% on the four datasets, respectively. Zhu et al. (2019) fine-tuned the pre-trained models (AlexNet, ResNet18, Xception, InceptionV3, DenseNet201, and NASNetLarge) for transfer training, and the results showed that the accuracy of all six models in the validation set reached 91%. Transfer learning is also used in the detection of agricultural pests and diseases: Chen et al. (2020) initialized the weight of the pre-trained network on the large labeled dataset ImageNet to classify and predict the images of rice leaf diseases and pests, with an average accuracy of 92.00%. Some scholars have also obtained good results in the detection of diseased leaves of tomatoes and grapes (Paymode and Malode, 2022). However, the application of transfer learning in the detection of rice seed vigor is still less studied. The main chemical components contained in different rice seeds are similar, so the NIR-HIS based depth model constructed can transfer the knowledge from the model to other varieties of rice seed models (He et al., 2019). In summary, the non-destructive assessment of rice seed vigor through near-infrared hyperspectral imaging technology holds significant value for the preservation of invaluable seed resources and pre-sowing screening. The utilization of transfer learning also presents the potential to conserve both time and resources, as there is no need to commence training an entirely novel model from scratch. This research amalgamates the domains of transfer learning and hyperspectral imaging technology, both at the forefront of modern computer science and agricultural science. It thereby offers intriguing avenues for further exploration and innovation.

In this study, the collected rice seed hyperspectral images were extracted from one-dimensional spectra, the one-dimensional spectrum was used as the input of the machine learning model to train the model, and then the knowledge was transferred by deep transfer to achieve the purpose of rice seed vigor detection. The main tasks of this study are to: (1) establish deep learning models for detecting rice seed vigor with high accuracy based on NIR-HIS for different varieties of rice seeds; (2) apply deep model knowledge transfer to the vigor classification of rice seeds of other varieties by deep transfer technology; (3) compare advantages and disadvantages of multiple transfer learning techniques in the detection of rice seed vigor; (4) use Grad-CAM++ to visualize the CNN model to identify the important wavelengths for seed vigor detection under different situations.

## 2 Materials and methods

### 2.1 Sample preparation and dataset description

In order to ensure the wide range of rice seed vigor, four rice varieties, Yongyou12, Yongyou1540, Suxiangjing100, Longjingyou1212, were selected for experimental analysis. The rice seeds used in this study were obtained from three different types, with a total of 7100 rice seed samples. The detailed distribution of the samples can be found in **Table 1**. We divided the dataset according to a 4:1:1 ratio. The rice seeds used in this study were provided by the College of Advanced Agricultural Sciences, Zhejiang A&F University, Lin’an, Zhejiang.

**Table 1:** The number of different varieties of seeds.

Variety	Type	Aging time	The number of seeds
Yongyou12	indica japonica hybrid rice	Not aged	600
		Aging 96h	600
		Aging192h	600
Yongyou1540	indica japonica hybrid rice	Not aged	600
		Aging 96h	600
		Aging192h	600
Suxiangjing100	regular japonica rice	Not aged	500
		Aging 96h	600
		Aging192h	600
Longjingyou1212	hybrid indica rice	Not aged	600
		Aging 96h	600
		Aging192h	600

Seeds aged under natural conditions are very rare, and the aging process is very long, which brings certain difficulties to the study of seed vigor. As a result, a growing number of studies have found that seeds can be artificially aged to mimic the natural aging process (Sena et al., 2017; Zhu et al., 2019; Yuan et al., 2022). In order to make a significant difference in seed vigor, the seeds were treated by high temperature and high humidity aging before the experiment. The rice seeds were placed in the LH-80 seed aging box (Top Cloud-agri Technology Co., Ltd., Hangzhou, China), the temperature was maintained at 45°C, the air moisture content was set to 100%, and the aging was 96 hours and 192 hours, respectively. Under high temperature and high humidity environment, a series of physiological and biochemical reactions such as membrane lipid peroxidation, soluble sugar and protein degradation, related gene expression disorders and nucleic acid degradation are accelerated, and the vitality of seeds is rapidly declining (Brar and Dudi, 2019).

In order to further verify the effect of artificial aging on seed vigor, standard germination experiments were performed on aged rice seeds (Seo et al., 2021). The seeds of the samples were placed on moist germination paper in the numbering order. The samples were subjected to a 14-day germination experiment under the conditions of 30°C light for 8 h and 20°C dark for 16 h every day. The experimental results showed that the number of not aged rice seeds after 96h aging was more than the number of not aged rice seeds after 192h aging, and the number of not aged seeds after 192h aging was the largest among the three aging gradients (not aged, aging 96h, aging 192h). In short, the seed vigor of rice after artificial aging was reduced, and the seed vigor decreased with the increase of aging time, which showed a linear negative correlation. The specific standard germination experiment results are shown in **Table 2**, where the non-viable rate is the ratio of the number of not aged seeds to the total number of seeds under this vigor gradient.

**Table 2:** The number of non-viable seeds of different varieties of seeds.

Variety	Aging time	Number	Non-viable rate
Yongyou12	Not aged	103	0.1717
	Aging 96h	291	0.4850
	Aging192h	424	0.7067
Yongyou1540	Not aged	112	0.1867
	Aging 96h	194	0.3233
	Aging192h	406	0.6767
Suxiangjing100	Not aged	93	0.1860
	Aging 96h	353	0.5883
	Aging192h	542	0.9033
Longjingyou1212	Not aged	117	0.1950
	Aging 96h	270	0.4500
	Aging192h	345	0.5750

### 2.2 Near-infrared hyperspectral image acquisition and correction

In this study, NIR-HIS was collected from four types of rice seeds. When acquiring hyperspectral images, the ambient room temperature was an average of 21°C and the average humidity was 73%. The ambient humidity and temperature at germination were similar to those when hyperspectral images were acquired. Images were acquired by the FX17 near-infrared hyperspectral camera (Specim, Spectral Imaging Ltd., Oulu, Finland) in conjunction with the hyperspectral image acquisition platform Lab Scanner (Specim, Spectral Imaging Ltd., Oulu, Finland). The FX17 near-infrared hyperspectral camera acquires a spectral range of 900-1700nm. Lab scanner is illuminated by two arrays of halogen lamps, each with a power of 35W. The sample stage on the stepper motor control Lab scanner pushes forward for imaging at a speed of 24.70mm/s and acquires spatial and spectral information of one row of data at a time; The sample stage surface is a high-gloss black surface with a white calibration whiteboard. The FX17 camera and Lab scanner are controlled by Lumo Scanner2020 (Specim, Spectral Imaging Ltd., Oulu, Finland) software. Hyperspectral images of 224 spectral channels were acquired per scan and then calibrated using the following equation:


(1) $$R=\frac{\text{I}-D}{W-D}\;\;$$

where R is the corrected image, I is the original image, W is the white reference image, and D is the dark reference image. The white reference image is obtained by shooting a white calibration whiteboard; Dark reference images are taken after the lens is completely covered with a lens cap. To avoid the instability of the system and the influence of noise in the spectral data on this study, the head and tail bands are removed and the wavelength range from 998 nm to 1631 nm was retained (Jin et al., 2022a). In this study, the average spectrum of each seed was obtained in the range of experimental accuracy: each seed in the hyperspectral image was treated as a region of interest (ROI), and the spectra of all cells in the ROI were averaged to obtain a spectral vector representing the seed sample.

### 2.3 Classification methods

#### 2.3.1 Logistic regression

Logistic regression is a classic machine learning algorithm. The idea of logistic regression is to first fit the decision boundary (not limited to linearity, but also polynomial), and then establish the probability of this boundary and the classification, so as to obtain the probability in the classification case (Jin et al., 2022b). Logistic regression typically uses the sigmoid function as the prediction function. Logistic regression has the advantage of avoiding inaccurate hypothetical distributions. In this study, L2 regularization was selected for LR and C was selected 1000.

#### 2.3.2 eXtreme Gradient Boosting

XGBoost (eXtreme Gradient Boosting) is a machine learning algorithm that demonstrates exceptional performance and serves as an extension of gradient boosting trees (Friedman, 2001). It enhances model performance by integrating multiple weak classifiers, resulting in outstanding predictive accuracy (Chen and Guestrin, 2016). XGBoost introduces regularization terms to control model complexity and incorporates penalty functions in the objective function for assessing model complexity, thereby preventing overfitting (Qiu et al., 2021). XGBoost has achieved remarkable success in various domains and tasks within the field of machine learning. During the training process of the XGBoost model, the parameter “reg_alpha” was set to 0.1, “reg_lambda” was set to 0.1, and “max_depth” was set to 2.

#### 2.3.3 Support vector machine

Support vector machine is a machine learning method proposed in the 90s of last century, which can be used for classification and regression tasks with superior performance and is widely used in research (Zhao et al., 2018; Cui et al., 2021). Support vector machines based on the principle of marginal maximization are used as a special linear classifier (Arun Kumar and Gopal, 2009). When the SVM is trained, it maps the data to a multidimensional space called decision space and finds the decision boundary in this space to complete the classification. When the data is linearly divisible, the decision boundary is a two-dimensional straight line; When the linearity is inseparable, the kernel function is used to map the feature data to the high-dimensional space, so that it becomes linearly separable in the high-dimensional space, and the decision boundary is a three-dimensional plane (Gopinath et al., 2020). SVM is an efficient nonlinear classifier that has strong robustness in processing classification problems under small sample conditions and can effectively handle high-dimensional data problems. In this study, the RBF and Poly kernel functions were selected and a grid search was performed to select the optimal parameters in ‘C’: [1,10,100,1000,10000], ‘gamma’: [1, 0.1, 0.01, 0.001].

#### 2.3.4 Convolutional neural network

Convolutional neural network is a special feedforward neural network integrated into convolution operations (Jin et al., 2022b), which not only has the characteristics that the neural network is composed of a large number of neurons, but also has excellent feature extraction and mapping capabilities because of convolution operations work with activation functions and normalization methods (Jin et al., 2022a). CNN algorithms essentially achieve input-to-output mapping by extracting features and reducing dimensionality (Voulodimos et al., 2018). CNNs consist of convolutional layers, pooling layers, and fully connected layers. The unit of the convolution layer is the feature graph, and each unit is related to the block of the previous feature graph by the filter group (Jin et al., 2018), the downsampling technique of the convolutional layer can capture the main spectral information (Xie et al., 2021). The main role of the pooling layer is dimensionality reduction. In the gradient-based optimization method, the ReLU activation function can reduce the effect of gradient vanishing, which has the advantage of preventing overfitting. The batch normalization layer (BN) can improve the efficiency of model training (Xie et al., 2021). Therefore, in the self-built CNN of this study, the ReLU activation function and BN are used in it. In addition, to further prevent overfitting, a dropout layer is added after the fully connected layer. Use the Adam optimizer for training and accelerated convergence. CNNs are widely used, have the ability to automatically learn features in images (Moujahid et al., 2022), and the trained CNN model can perform transfer learning in different tasks, which is where this institute especially needs it. However, the generalization ability of CNN is weak. In order to comprehensively explore the generalization ability of CNN, researchers (Sun et al., 2021) had set up three different experimental conditions for evaluation. The experimental results demonstrated the weak generalization ability of CNN-based methods. Therefore, it is of great significance to enhance the generalization ability of CNN through transfer learning.

**Figure 1:**
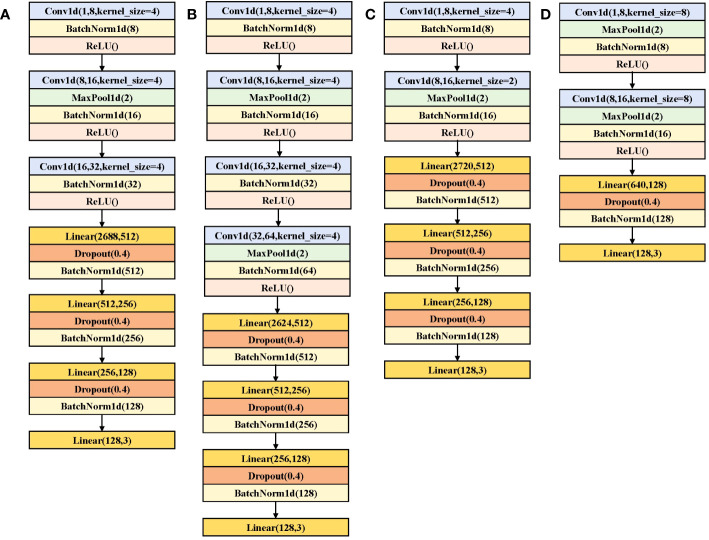
CNN structure diagram for the detection of rice seed vigor by **(A)** Yongyou12, **(B)** Yongyou1540, **(C)** Suxiangjing100, and **(D)** Longjingyou1212.

In this study, four CNN with different structures were designed for four types of rice seeds, with a maximum of 4 layers and a minimum of 2 layers, as shown in **Figure 1**. Convolution kernels of 1 × 4 were used in the four CNN to extract features hidden in spectral vectors. Also, BN and ReLU activation functions were used after each convolution to reduce the risk of overfitting and speed up the convergence process. A maxpooling layer with a kernel size of 1 × 2 was added after the partial convolutional layer to reduce the feature dimension. The parameter of the Dropout layer was set to 0.4. Different batch size and learning rates were set for the four CNN models, 32,0.005; 32,0.001; 32,0.0005; and 32,0.0005. Stride is all set to 1.

### 2.4 Transfer learning strategy

**Figure 2:**
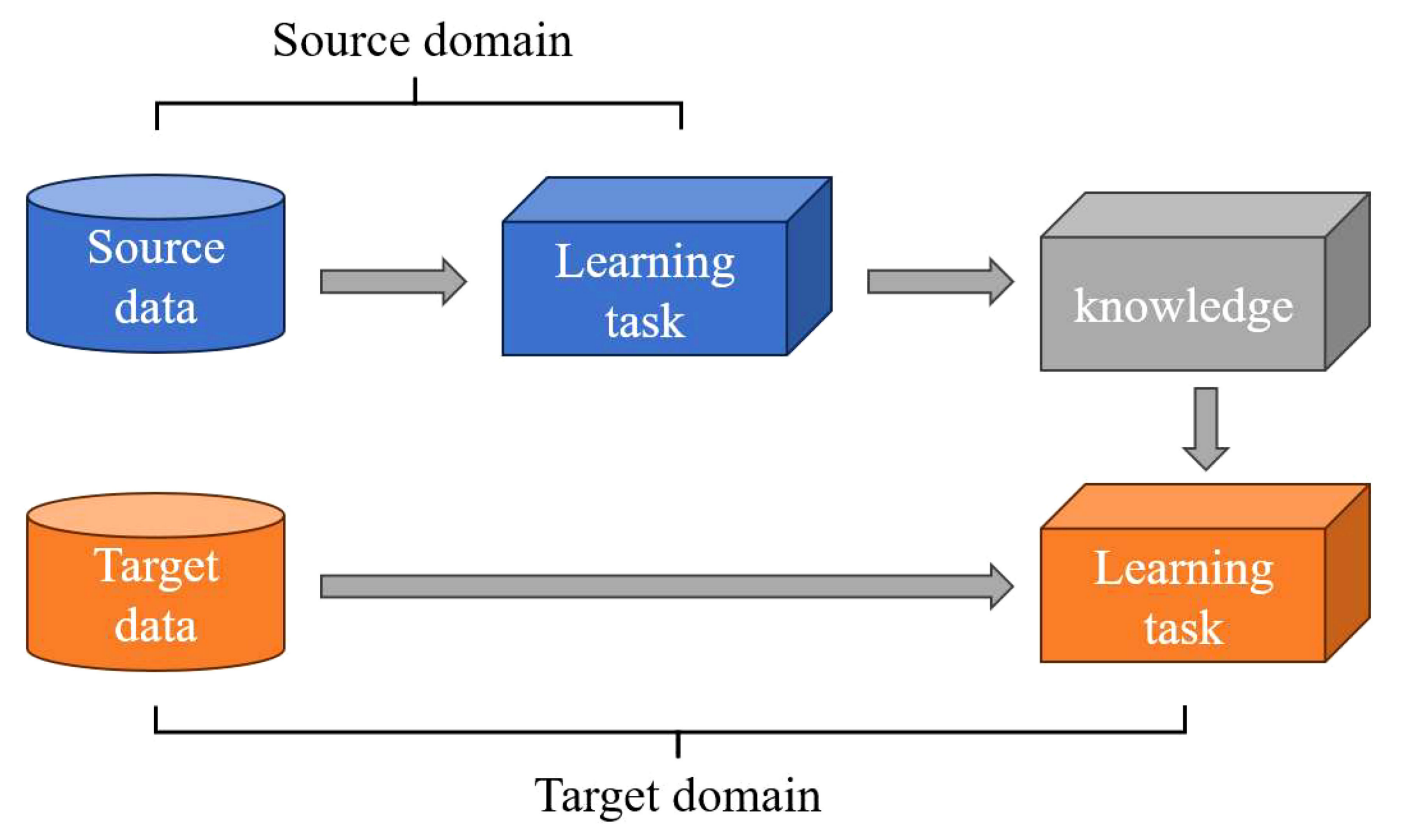
Transfer learning process.

Transfer learning is an emerging machine learning tool that is considered an important small-sample learning method (Xiao et al., 2019; She et al., 2021), and it has been proposed to alleviate the need for sufficient training data for the model by transferring the available knowledge in the relevant source domain to the target domain (Pan and Yang, 2010; Xie et al., 2021). For the detection of rice seed vigor in the agricultural field, transfer learning can greatly save time and resources, improve the generalization ability of the model, and also have obvious advantages in solving the problem of data scarcity. Transfer learning is expressed by the formula: let the source domain be $D_{s}={\left\{x_{i},y_{i}\right\}}_{i}^{N_{s}}$ , and the target domain is $D_{t}={\left\{x_{i},y_{i}\right\}}_{i}^{N_{t}}$ , where $x_{i}$ and $\;y_{i}$ represent data samples and their corresponding labels, respectively. Given the $D_{s}$ and learning task $T_{s}$ , $D_{t}$ , and learning task $T_{t}$ , improve the performance of the prediction function $f_{t}\left(\cdot \right)\;$ in the $D_{t}$ by acquiring knowledge in the $D_{s}$ and $T_{s}$ , $D_{s}\neq D_{t}$ , $T_{s}\neq T_{t}$ . The transfer process is shown in **Figure 2**.

#### 2.4.1 Fine-tuning

Fine-tuning is a simple and versatile strategy in transfer learning. The pre-trained depth models has been adapted to new tasks (Cetinic et al., 2018). It can effectively reduce modeling costs. Fine-tuning is: there is a pre-trained model $Model_{pre}$ that has been trained on large-scale data with a set of parameters $\theta_{pre}$ , and now we have a specific task that aims to minimize a specific loss function $L_{\theta }$ , where θ represents the parameters of the model. The goal of fine-tuning is to fine-tune the parameters of a pre-trained model so that it performs well on specific tasks. The process of fine-tuning can be expressed as the following optimization problem: $min_{L_{\theta }}$ . The initial layer of the CNN retains abstract, generic features, while the top layer retains more specific features related to the task (Vrbancic and Podgorelec, 2020). Considering the characteristics of the above CNN, there are two main ideas for fine-tuning: (1) only adjust the last few fully connected layers; (2) Adjust all network layers (Zhen et al., 2017). The outcome of knowledge transfer depends on the similarity between the trained CNN and the target task we want to transfer knowledge. In this study, the distribution and features between the source domain data and the target domain data were similar, and the convolutional layer before the fully connected layer may have extracted the important feature information of the seed spectrum and can be reused in the target domain (Wu et al., 2021), so the parameters of each layer before the pre-trained CNN fully connected layer were frozen, and the last few fully connected layers were fine-tuned.

#### 2.4.2 MixStyle

As the role of deep transfer is discovered by more and more people, more deep transfer strategies are proposed, such as BNM (Cui et al., 2020), MCC (Jin et al., 2020) and V-REx (Krueger et al., 2021) and so on. In order to cope with the data distribution problem in different domains, the above deep transfer methods can be roughly divided into domain adaptation and domain generalization. Domain adaptation refers to the process of transferring information to one or more source domains in order to improve the learning performance of target learners (Pan and Yang, 2010). Domain generalization aims to improve the generalization ability of models by leveraging useful information from multidomain data (Du et al., 2022). In view of the relatively similar spectral data distribution between rice varieties, MixStyle belonging to domain generalization was selected in this study (Zhou et al., 2021).

The method was proposed by Zhou et al. in 2021, a novel method based on probabilistically mixing instance level feature statistics of training samples across source domains. The method mixes the feature statistics of two instances with a random convex weight to simulate the new style.

A batch of data is sampled from each of the $domain_{i}$ and the $domain_{j}$ , $x_{i}$ and the $x_{j}$ form $x=\left[x_{i},x_{j}\right]$ , swap the position of the batch to obtain $\tilde{x}$ , and then shuffle each batch along the batch dimension. After shuffling, MixStyle computes the mixed feature statistics by:


(2) $$\gamma_{mix}=\lambda \sigma \left(x\right)+\left(1-\lambda \right)\sigma \left(\tilde{x}\right)$$

(3) $$\beta_{mix}=\lambda \mu \left(x\right)+\left(1-\lambda \right)\mu \left(\tilde{x}\right)$$

where $\lambda \in {\mathbb{R}}^{B}$ are instance-wise weights sampled from the Beta distribution, λ ∼ Beta(α, α)  with α∈(0,∞) being a hyper-parameter, we set α  to 0.1. Finally, the mixed feature statistics are applied to the style-normalized x :


(4) $$MixStyle\left(x\right)=\gamma_{mix}\frac{x-\mu \left(x\right)}{\sigma \left(x\right)}+\beta_{mix}$$

### 2.5 Visualization method

CNNs are a “black box” model in the field of deep learning, and in order to make it easier to interpret CNN results, Gradient-weighted Class Activation Mapping++ (Grad-CAM++) was used to visualize CNN models in this study. Grad-CAM++ is a generalized approach based on Grad-CAM that better provides visualization results for CNNs (Cai et al., 2023). It uses the gradient of any target concept flowing into the final convolutional layer to generate a coarse localization map that highlights important areas of the image for predicting the concept (Moujahid et al., 2022). It can be adapted to any CNN model (Afify et al., 2023).

### 2.6 Model evaluation and software

**Figure 3:**
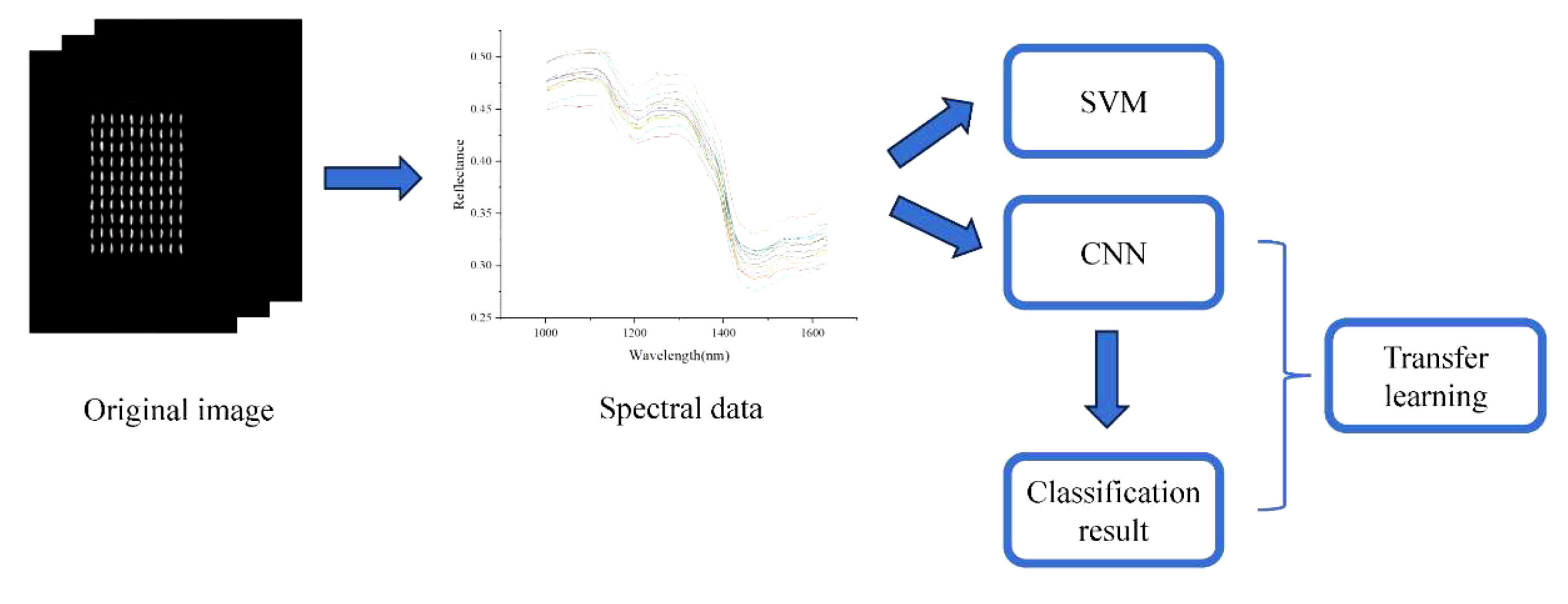
Experimental flow chart.

**Figure 3** is the experimental flow chart of this article. using a 1D spectrum with 181 features after noise reduction as input. This study used classification accuracy to evaluate the performance of the model. The SVM, LR and XGBosst used in this study was based on Python 3.8 and scikit learn 1.0.2; the deep learning model was built on pytorch 1.10.2. The deep model was trained using the NVIDIA GeForce RTX 3060 Laptop GPU for acceleration. Data analysis was performed on computers configured with Intel (R) Core (TM) i7-11800H (2.3GHz) and 16G RAM. All data analysis was performed on Windows 10.

## 3 Results

### 3.1 Spectral analysis

**Figure 4:**
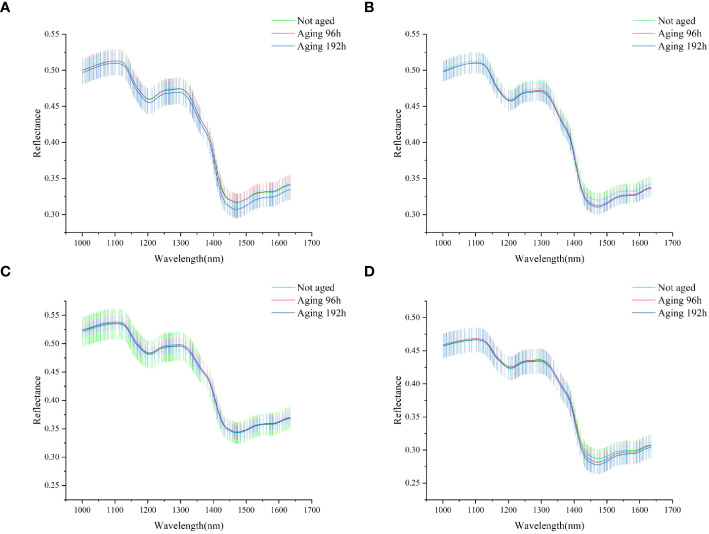
The average spectra with the standard deviation of four rice seeds: **(A)** Yongyou12; **(B)** Yongyou1540; **(C)** Suxiangjing100; **(D)** Longjingyou1212.

In this study, all the spectral data collected were averaged to obtain the average spectra and standard deviation of four types of rice seeds, as shown in **Figure 4**.

In general, the shape of the spectrum and the location of the peak of the four types of rice seeds were very similar, which indicated that there may be more common characteristics between the four types of rice seeds, which was conducive to the transfer learning. The spectral difference between Yongyou12 rice seeds under different aging gradients was the largest among the four types of rice seeds, and the wavelength differences were most obvious at 1150-1300 nm and 1400-1650 nm. Suxiangjing100 rice seeds had minimal spectral differences. The wavelengths of the four types of rice seeds with different vigor spectra were concentrated at 1400-1650 nm, and the band could be attributed to the first overtone of amide A (N–H stretch), which might be the critical band for protein detection (Wu et al., 2019). The 1100 nm band were caused by the second overtone of carbohydrates (C–H stretch) (Wu et al., 2021). The peak near 1300 nm was reported to be associated with the combinations of the first overtone of amide B (N–H stretch) and the fundamental vibrations of amide II and III (C–N stretch and N–H in-plane bend) (Daszykowski et al., 2008).

Standard deviation is a measure of how dispersed or dispersed a set of data is. The introduction of standard deviation helps to understand the degree of change in multiple spectral curves. The small standard deviation indicates that their spectral curves are relatively close and have similar fluctuation patterns. As can be seen from **Figure 4**, the standard deviation of all four types of rice seeds in the 1350-1400 nm band was small.

### 3.2 Classification results on source dataset

In this study, LR, XGBoost, SVM and CNN were used to determine the vigor of four types of rice seeds with different aging gradients. In our investigation, True Positives (TP) were defined as instances where the model accurately categorizes not aged seed as not aged. True Negatives (TN) referred to cases where the model correctly classifies seeds aging 96 hours and 192 hours as not aged. False Positives (FP) represented erroneous classifications where the model incorrectly labels some seeds aging 96 hours or 192 hours as not aged. False Negatives (FN) denoted situations where the model incorrectly classifies some not aged seeds as aging 96 hours or 192 hours. The same definitions applied to the cases of seeds aging 96 hours and 192 hours as well. The parameters for LR, XGBoost and SVM were described in 2.3.1, 2.3.2 and 2.3.3, respectively. The number of CNN training epochs was 2000, we selected the best round of results as a showcase. The models started fitting around 1500 rounds.

The categorization of rice seed vigor among the four distinct types using the LR model resulted in suboptimal outcomes, with an accuracy rate of approximately 65.00%. Notably, LR demonstrated better performance in classifying Yongyou1540 rice seed vigor, achieving a commendable test set accuracy of 72.33%. However, LR’s performance was inferior to that of SVM and CNN. This could be attributed to LR being a linear model, which may not fit the data well for complex nonlinear relationships, such as one-dimensional spectral data. Furthermore, the small sample size may have also contributed to the lower performance.

The experimental results indicated that the performance of XGBoost was rather disappointing, as it exhibited the poorest overall performance among all classifiers. The accuracy on both the validation and test sets remained around 60.00%, with the occurrence of overfitting.

When using SVM and CNN models for vigor detection, there were slight instances of overfitting in the models. This phenomenon could potentially be linked to the relatively limited quantity of training samples available. Both SVM and CNN consistently achieved an accuracy rate surpassing 90% when applied to the training dataset. Notably, the Yongyou12 rice seed variety demonstrated the most congruent accuracy levels between SVM and CNN, exhibiting remarkable stability across both classifiers.

SVM and CNN exhibited remarkable proficiency when employed to categorize Yongyou1540 rice seeds. The classification accuracy across the training, validation, and test datasets demonstrated notable consistency, with CNN achieving the highest accuracy among the four seed types on the test set, reaching an impressive 90.33%. However, it is worth noting that the classifier’s performance was comparatively less impressive when dealing with Suxiangjing100 and Longjingyou1212, falling short of the performance demonstrated on Yongyou12 and Yongyou1540. Nevertheless, the overall classification accuracy remained commendable.

In summary, CNN consistently outperformed SVM in terms of classification accuracy, owing to the convolution operation’s ability to extract more intricate feature information from the abundant spectral data. The architectural framework of the CNN model employed is depicted in **Figure 1**, and detailed classification accuracy metrics can be found in **Table 3**.

**Table 3:** The results of detection of seeds vigor.

Variety	Model	Accuracy(%)		
		Training	Validation	Test
Yongyou12	LR	69.92	65.00	65.67
	XGBoost	93.08	61.00	63.00
	SVC	94.58	87.00	87.33
	CNN	94.25	87.00	87.67
Yongyou1540	LR	71.67	70.67	72.33
	XGBoost	94.25	60.67	64.33
	SVC	96.33	91.33	89.67
	CNN	99.92	88.67	90.33
Suxiangjing100	LR	64.99	62.30	60.65
	XGBoost	95.74	60.12	60.84
	SVC	91.09	84.10	83.45
	CNN	99.38	83.75	86.27
Longjingyou1212	LR	63.33	63.00	64.67
	XGBoost	93.17	59.00	61.00
	SVC	90.17	83.00	81.67
	CNN	95.17	82.00	84.67

### 3.3 Results of fine-tuning

In order to verify the feasibility of transfer learning in rice seed vigor detection, this study used the most common fine-tuning in the field of transfer learning to adjust the pre-training model to adapt it to the vigor detection of other rice seeds. Our main idea was to design 4 different CNN models for specific rice seeds, and then fine-tuned the CNN models, frozen the layer before the fully connected layer, adjusted the fully connected layer, and transferred to the vigor identification of the other three rice seeds. The feasibility of fine-tuning was verified by taking the accuracy rate as the criterion, and the specific experimental results are shown in **Table 4**.

**Table 4:** The results of detection of seeds vigor using fine-tuning.

Variety	Fine-tuning to	Accuracy (%)			Comparison with Table 3 (%)		
		Training	Validation	Test	Training	Validation	Test
Yongyou12	Yongyou1540	96.58	87.67	87.67	-3.34	-1.00	-2.66
	Suxiangjing100	95.32	80.57	82.04	-4.06	-3.18	-4.23
	Longjingyou1212	84.25	80.60	82.33	-10.92	-1.40	-2.34
Yongyou1540	Yongyou12	97.08	82.67	87.33	+2.83	-4.33	-0.34
	Suxiangjing100	95.50	77.39	81.28	-3.88	-6.36	-4.99
	Longjingyou1212	95.42	75.33	77.00	+0.25	-6.67	-7.67
Suxiangjing100	Yongyou12	94.92	76.67	82.33	+0.34	-10.33	-5.00
	Yongyou1540	95.00	83.67	83.00	-4.92	-5.00	-7.33
	Longjingyou1212	97.17	71.33	73.33	+2.00	-10.67	-11.34
Longjingyou1212	Yongyou12	92.50	86.67	90.00	-1.75	-0.33	+2.33
	Yongyou1540	92.58	89.67	89.33	-7.34	+1.00	-1.00
	Suxiangjing100	90.56	84.10	85.56	-8.82	+0.35	-0.71

From **Table 4**, it can be concluded that the overfitting phenomenon of each model after fine-tuning had improved compared with before fine-tuning. The CNN model fine-tuning of Longjingyou1212 performs best when used for other rice seed vigor and was closest to the accuracy of each CNN model before fine-tuning. When it was transferred to the detection of Yongyou12 rice seed vigor, the accuracy rate was improved by 2.33% compared with the original model, reaching 90.00%. When the CNN model fine-tuning of Yongyou12 and Yongyou1540 was used for other rice seed vigor detection, the performance was average, but the accuracy was similar to that before fine-tuning. The CNN model fine-tuning results of Suxiangjing100 were poor and there was a large negative transfer (Wu et al., 2021), which may be due to the fact that compared with other rice varieties, the spectrum of Suxiangjing100 had its own unique characteristics, that was, the difference between the source domain data and the target domain data was large, resulting in poor results.

The results in **Table 4** shows that the overall fine-tuning can meet the requirements of model generalization, but there was a certain gap between the accuracy rate and the accuracy of each model before fine-tuning, and this gap was most obvious in the micro-sculpting of Suxiangjing100. When there were large differences in the data sets, fine-tuning may not achieve the desired effect. Therefore, we needed to find a more effective transfer learning strategy.

### 3.4 Results of MixStyle

MixStyle is designed to normalize CNN training by perturbing the style information of the training instance in the source domain. It is designed to be plugged between CNN layers as a plug-and-play module. The essence of the MixStyle approach lies in utilizing a CNN as a feature extractor. The process begins by passing the input sample through the feature extractor, which generates a specific feature map. Next, multiple style samples are inputted into the feature extractor to generate corresponding style feature maps. During the training process, a hybrid style feature map is created for classification prediction. This is achieved through linear interpolation and synthesis of the style feature maps. This blending of style features enables the model to capture a diverse range of styles and improve its ability to classify different samples effectively. The potential superiority of MixStyle arises from its ability to synthesize “new” styles, thereby regularizing the network to become more resilient to domain shifts. When using MixStyle for transfer learning, the Aadm optimizer was used, and the learning rate of all models was set between [0.0005, 0.000001]. To mitigate overfitting, L2 regularization was used, set to 0.0005. The training process consisted of 20,000 epochs, and the best result was selected.

MixStyle involves mixing feature statistics for two instances with random convex weights to simulate the new style. A reference batch is generated by swapping the locations of instances from different domains and applying a shuffle operation. In this study, the training sets of two types of rice seeds were mixed, and the vigor prediction was carried out with one of the rice seeds as the target domain. We added the MixStyle layer after the last convolutional layer. The model structure used is shown in **Figure 1**. Taking the transfer of Yongyou12 rice seed knowledge to Yongyou1540 as an example, Yongyou12 rice seed data is the source domain and Yongyou1540 rice seed data is the target domain. During model training, the Yongyou12 rice seed training set data and the Yongyou1540 rice seed training set data were extracted by the **Figure 1A** model, then mixed by MixStyle, and finally the rice seeds in the source domain were predicted. After the model training is completed, the target domain seed is used as the validation set and the test set to test the model performance, that is, the model is applied to the prediction of the target domain Yongyou1540 rice seed vigor.

As can be seen from **Table 5**, after MixStyle mixing, the performance of each model in the training set and the validation set was better, and the accuracy rate was generally higher than 80.00%. Among them, when the Yongyou12 rice seed data was used as the source domain, the performance of the model was the best. When mixed with other varieties, the accuracy rate was 86.00%, 84.07% and 81.33% on the test set, respectively. When Yongyou12 knowledge transfer was used to predict Yongyou1540 rice seed vigor, the model had the best performance, with 94.47%, 90.00% and 86.00% accuracy on the training set, validation set and test set, respectively, and the model performance was also better when Longjingyou1212 was used as the source domain for transfer. In conclusion, MixStyle can significantly improve the generalization performance of CNN in detecting rice seed vigor.

**Table 5:** The results of detection of seeds vigor using MixStyle.

Variety	Mixstyle with	Accuracy (%)			Comparison with Table 3 (%)		
		Training	Validation	Test	Training	Validation	Test
Yongyou12	Yongyou1540	94.50	90.00	86.00	0.22	1.33	-4.33
	Suxiangjing100	89.75	80.33	84.00	-9.63	-3.42	-2.27
	Longjingyou1212	96.50	85.00	81.33	1.33	3.00	-3.34
Yongyou1540	Yongyou12	94.92	82.33	83.33	0.67	-4.67	-4.34
	Suxiangjing100	89.42	82.33	77.33	-9.96	-1.42	-8.94
	Longjingyou1212	91.58	79.67	78.33	-3.59	-2.33	-6.34
Suxiangjing100	Yongyou12	86.33	80.67	79.67	-7.92	-6.33	-8.00
	Yongyou1540	84.25	82.00	83.33	-15.67	-6.67	-7.00
	Longjingyou1212	88.75	79.33	76.00	-6.42	-2.67	-8.67
Longjingyou1212	Yongyou12	93.25	81.33	80.67	-1.00	-5.67	-7.00
	Yongyou1540	94.67	84.67	83.67	-5.25	-4.00	-6.66
	Suxiangjing100	93.75	80.33	79.33	-5.63	-3.42	-6.94

### 3.5 Model visualization

To demonstrate the model training process, we took the example of Yongyou1540 rice seeds. In **Figures S1** (in **Supplementary Materials**), we showcased the variations in loss and accuracy during the training process of the original CNN model, fine-tuned CNN model, and the CNN model with MixStyle incorporated. These figures clearly depict a consistent decrease in loss and a progressive increase in accuracy, ultimately converging to indicate a good fit.

**Figure 5:**
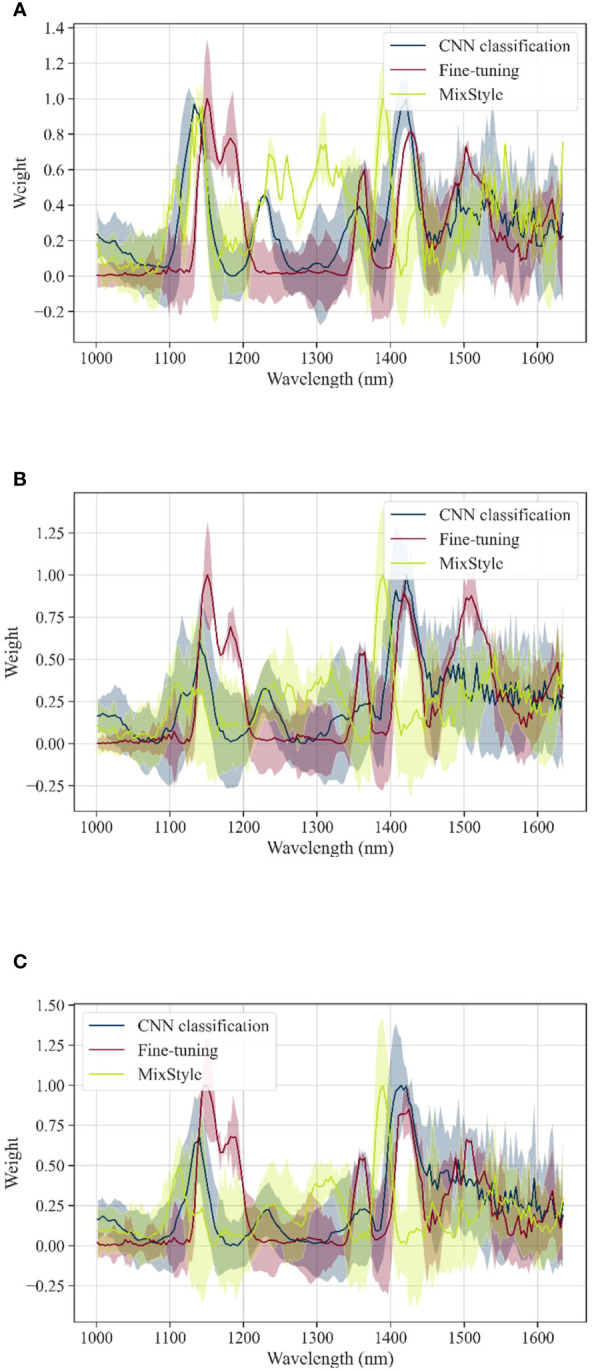
Grad-CAM++ of each vigor gradient of CNN: Yongyou12 transfer to Yongyou1540: **(A)** the weights of not aged; **(B)** the weights of aging 96h; **(C)** the weights of aging 192h.

**Figure 5** shows the visualization of each band when using CNN classification after transferring Yongyou12 to Yongyou1540 using Fine-tuning and MixStyle, with three top-to-bottom Grad-CAM++ plots of (A) not aged, (B) aging 96h, and (C) aging 192h. Among them, the curve represents the weight of each spectral wavelength on the model, and the higher the weight of the wavelength, the greater the contribution to the model classification result. The shaded area with the same color as the curve represents the corresponding standard deviation of the weight value for each wavelength. The weight values and standard deviations of each wavelength of the CNN using Fine-tuning and MixStyle were compared to the weight values and standard deviations of each wavelength of the original CNN. It could be seen from the figure that for the vigor classification of Yongyou12 knowledge transferred for Yongyou1540 rice seeds, the weight distribution of CNNs after transfer was similar to that of the original CNN classification, and the wavelengths with larger weights were mainly distributed in the 1100-1200 nm and 1400-1450 nm ranges. Compared with the weight distribution of CNNs after fine-tuning, the weight distribution of CNNs after knowledge transfer through MixStyle was more similar to that of the original CNNs, and the weights assigned by CNNs after Fine-tuning were higher in the wavelength range of 1450-1550 nm, which was far from the region where the original CNN assigns weights. **Figures S2, S3** shows the knowledge transfer of Yongyou12 rice seeds for Suxiangjing100 and Longjingyou1212 rice seed vigor classification, respectively, with weight distribution similar to **Figure 5**, with higher weighted regions concentrated in the 1100-1200 nm and 1400-1450 nm intervals. **Figures S4–S6** is the CNN visualization result of Yongyou1540 knowledge transfer for the vigor classification of other rice varieties, and it can be seen that the weight assigned in the wavelength range of 1400-1450 nm was higher, and the weight distribution of CNN after MixStyle knowledge transfer was closer to the weight distribution of the Fine-tuning. The wavelengths with higher weights in were also mainly distributed in the 1100-1200 nm and 1400-1450 nm ranges, and the transfer ability of MixStlye was similar to Fine-tuning from the wavelength weight distribution in **Figures S7–S9**. The weight distribution of CNN after knowledge transfer of Longjingyou1212 is shown in **Figures S10–S12**, the original CNN assigned higher weights in the wavelength range of 1100-1150 nm, but the CNNs after transfer learning did not assign higher weights in this interval. In the remaining interval, the weights assigned by the transfer-learned CNN and the original CNN were extremely similar, so when Longjingyou1212 was used for the vigor classification of Yongyou12, Yongyou1540 and Suxiangjing100, the classification accuracy was also higher in all cases.

## 4 Discussion

The identification of rice seed vigor plays a crucial role in improving germination rates, increasing yields, reducing pests and diseases, and optimizing resource utilization. In this study, near-infrared hyperspectral imaging was utilized to detect the vigor of artificially aged rice seeds. Furthermore, transfer learning was employed to transfer knowledge and enhance the model’s generalization ability. The findings indicate that NIR-HIS is an effective method for detecting rice seed vigor. Moreover, employing transfer learning significantly improves the model’s ability to generalize. Previous research mainly focused on training models from scratch (Jin et al., 2022a; Wang et al., 2022), which is time-consuming and labor-intensive, especially for deep models that require a substantial number of samples. In this study, we adopted traditional machine learning methods and developed custom CNN to discriminate rice seed vigor. Transfer learning was then utilized to transfer knowledge to pre-trained models, enabling rice seed vigor detection across different varieties. While many scholars have explored transfer learning (Zhen et al., 2017; Cetinic et al., 2018; Wu et al., 2021), their approaches were limited to basic Fine-tuning, which often yielded unsatisfactory results. In contrast, our study not only employed Fine-tuning but also utilized the MixStyle transfer strategy to transfer knowledge between different rice seeds. The results demonstrate that the deep model outperformed the traditional machine learning model in terms of classification performance, highlighting the superior generalization ability offered by the deep transfer strategy. In the future, we plan to include more rice varieties in the study, with more generalized models adapted to more rice variety vigor detection. In addition, crop pest and disease data are also difficult to obtain, so in the future, we will also apply near-infrared hyperspectral imaging technology and transfer learning to pest detection to detect pests and diseases early and take corresponding control measures.

## 5 Conclusion

In this study, the potential of near-infrared hyperspectral imaging in seed vigor detection was discussed, and remarkable results were achieved by combining transfer learning methods, and the CNN model of Yongyou12 classifies the vigor of Yongyou1540, Suxiangjing100 and Longjingyou1212 through MixStyle transfer knowledge, and the accuracy reaches 90.00%, 80.33% and 85.00%, respectively. NIR hyperspectral imaging is a non-invasive means to capture the absorption and reflection properties of rice seeds at different wavelengths, providing useful information about the internal chemical composition of the seeds. Transfer learning effectively improves the generalization performance of the model by sharing similar spectral characteristics between different seed varieties, thereby improving the performance and robustness of the model. In the future, we plan to apply near-infrared hyperspectral imaging techniques and transfer learning to the field of crop diseases and pests. This study provides a viable approach to detect rice seed vigor and enhance the generalization ability of the model in situations with limited sample sizes. It also reduces the cost of seed testing, thereby contributing to the promotion of sustainability in agricultural products. These advancements hold positive and long-term implications for the agricultural sector and food production.

## Data availability statement

The raw data supporting the conclusions of this article will be made available by the authors, without undue reservation.

## Author contributions

HQ: Conceptualization, Funding acquisition, Project administration, Resources, Writing – review & editing. ZH: Conceptualization, Formal Analysis, Investigation, Methodology, Software, Writing – original draft, Writing – review & editing. ZS: Data curation, Writing – review & editing. QT: Data curation, Writing – review & editing. GZ: Investigation, Resources, Writing – review & editing. XZ: Methodology, Writing – review & editing. CZ: Conceptualization, Supervision, Validation, Writing – review & editing.
