# Supplementary material for: Rice seed vigor detection based on near-infrared hyperspectral imaging and deep transfer learning
Source: Front Plant Sci. 2023 Oct 23;14:1283921. doi: 10.3389/fpls.2023.1283921 (PMC10627025; doi:10.3389/fpls.2023.1283921)
Supplement: Supplementary file 1 [file Presentation_1.pdf]

Supplementary Material

Rice Seed Vigor Detection Based on Near-Infrared Hyperspectral Imaging and Deep Transfer Learning

Hengnian Qi†, Zihong Huang†, Zeyu Sun, Qizhe Tang, Guangwu Zhao, Xuhua Zhu, Chu Zhang*

*** Correspondence:** Chu Zhang: chuzh@zjhu.edu.cn

1 Supplementary Figures

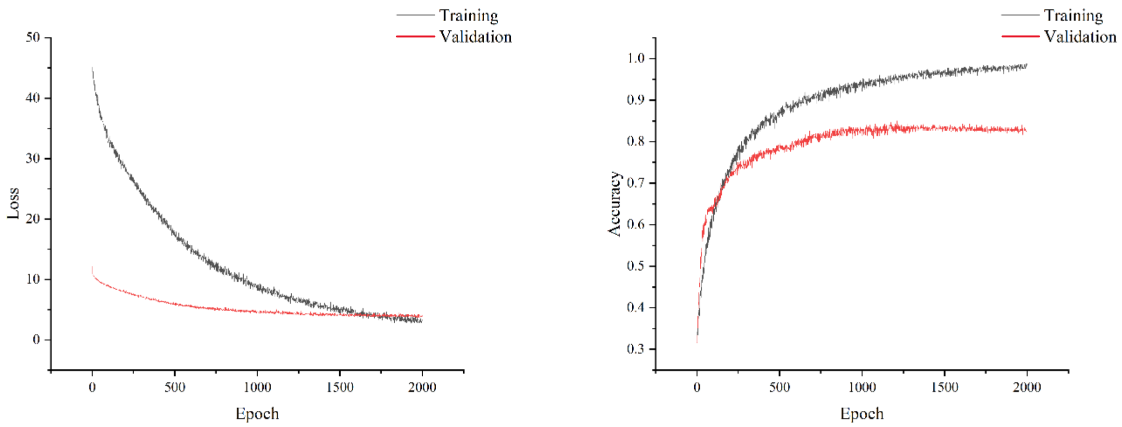

(A)

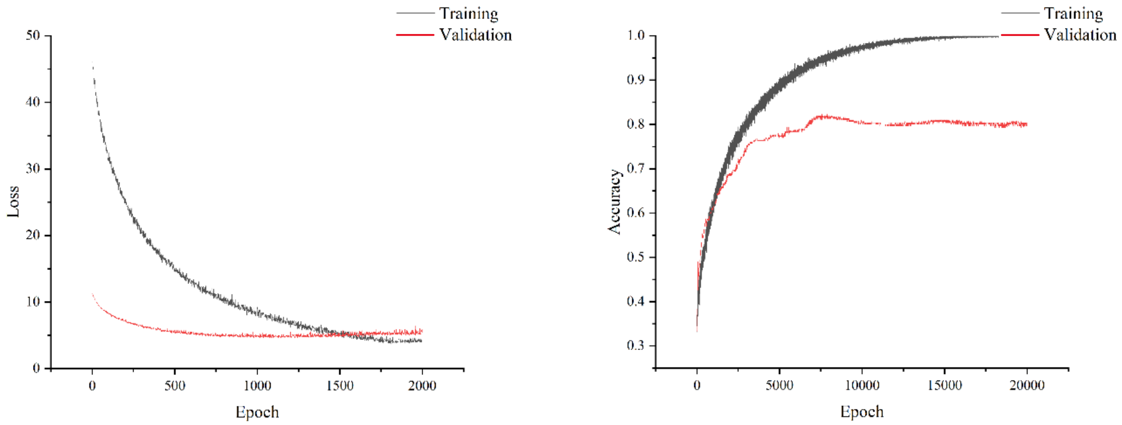

(B)

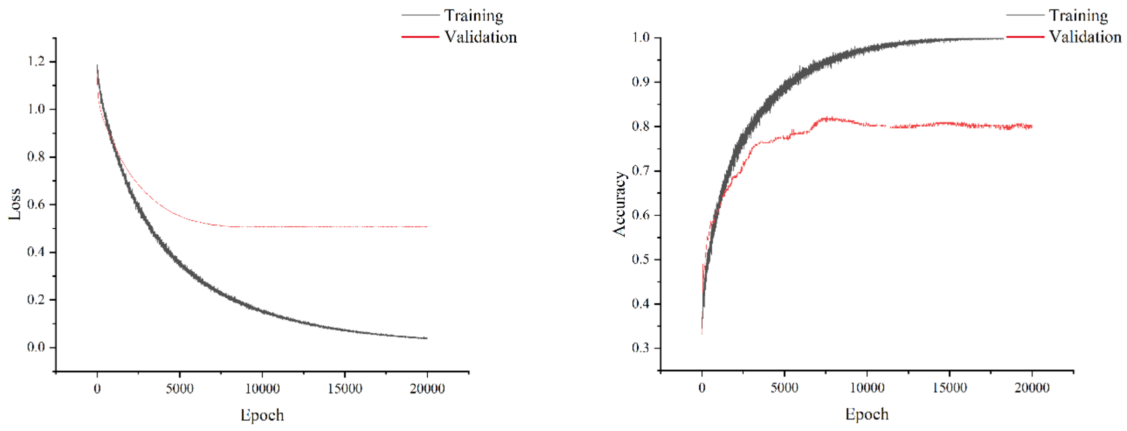

(C)

Figure. S1. The loss and accuracy curve of CNN model of Yongyou1540 rice seeds: (A) MixStyle with Yongyou12; (B) MixStyle with Suxiangjing100; (C) MixStyle with Longjingyou1212.

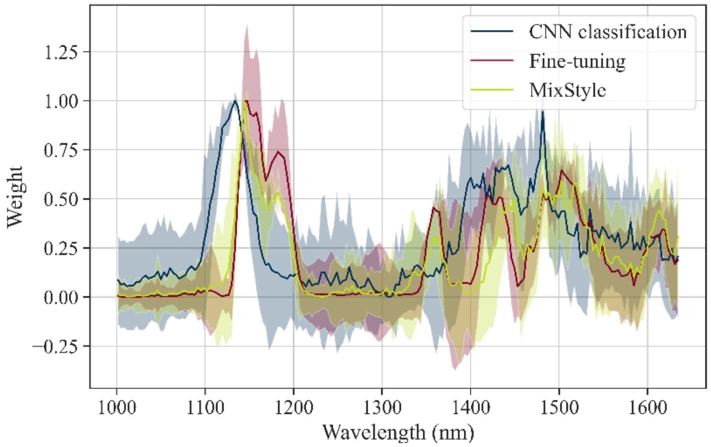

(A)

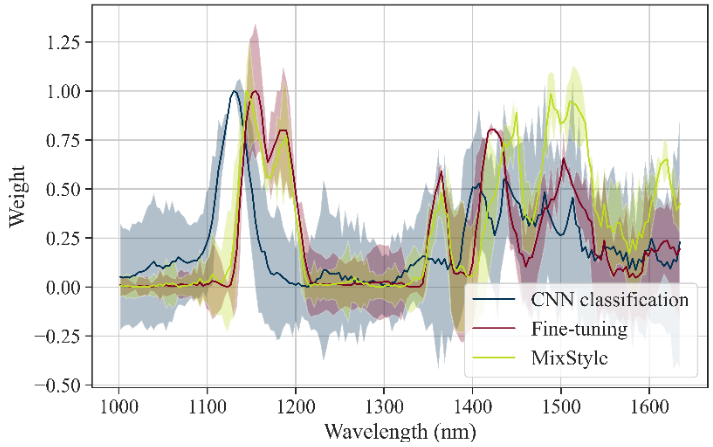

(B)

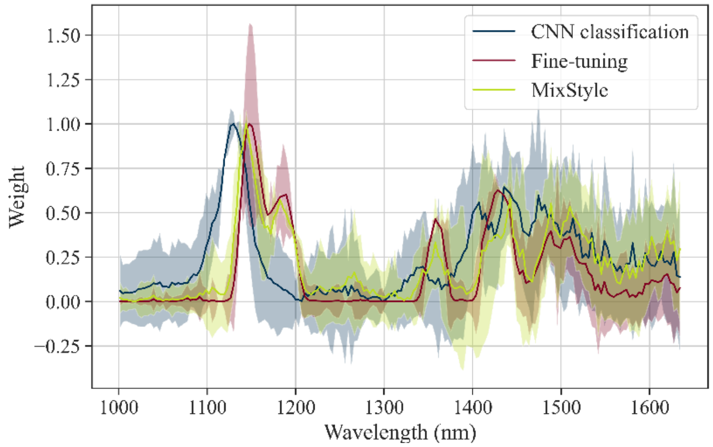

(C)

Figure. S2. Grad-CAM++ of each vigor gradient of CNN: Yongyou12 transfer to Suxiangjing100: (A) the weights of not aged; (B) the weights of aging 96h; (C) the weights of aging 192h.

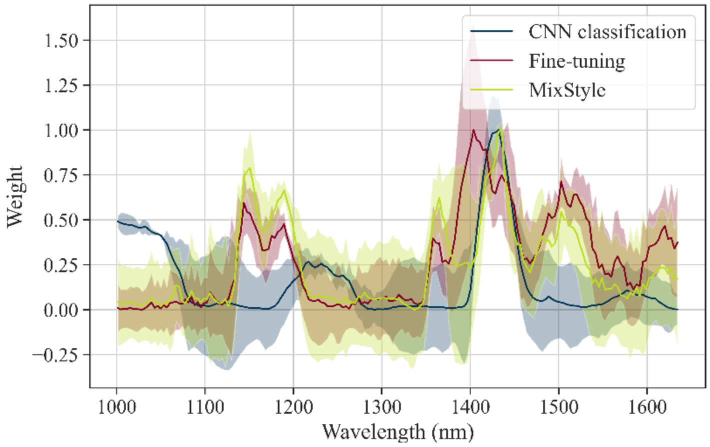
CNN classification
Fine-tuning
MixStyle
Weight
1.50
1.25
1.00
0.75
0.50
0.25
0.00
−0.25
1000
1100
1200
1300
1400
1500
1600
Wavelength (nm)

(A)

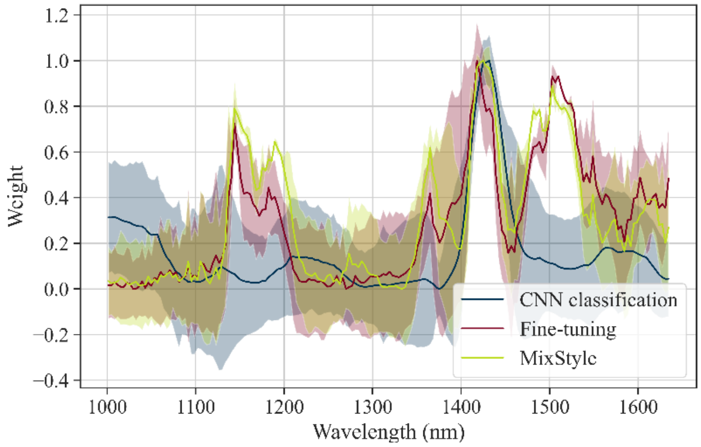
CNN classification
Fine-tuning
MixStyle
Weight
1.2
1.0
0.8
0.6
0.4
0.2
0.0
−0.2
−0.4
1000
1100
1200
1300
1400
1500
1600
Wavelength (nm)

(B)

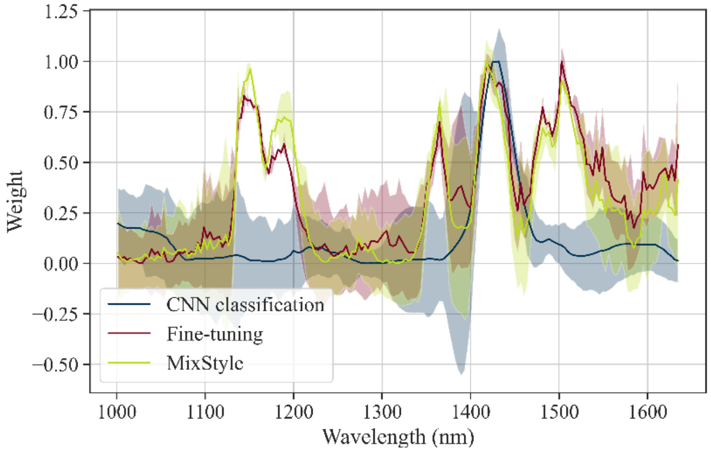

(C)

Figure. S3. Grad-CAM++ of each vigor gradient of CNN: Yongyou12 transfer to Longjingyou1212: (A) the weights of not aged; (B) the weights of aging 96h; (C) the weights of aging 192h..

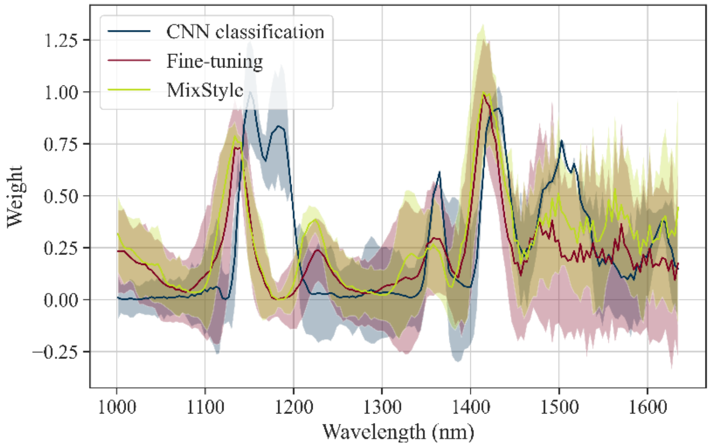

(A)

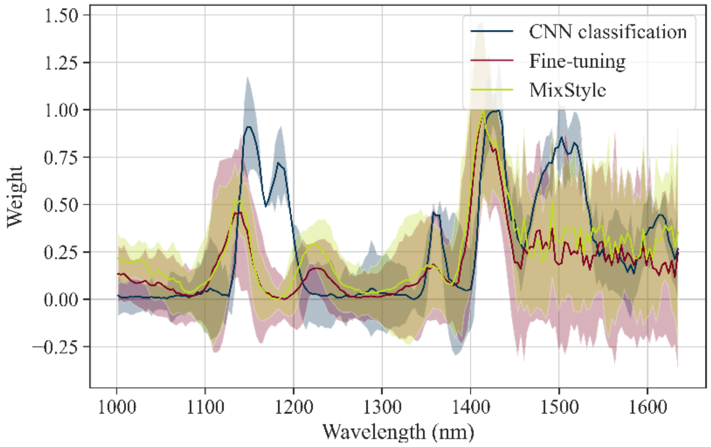

(B)

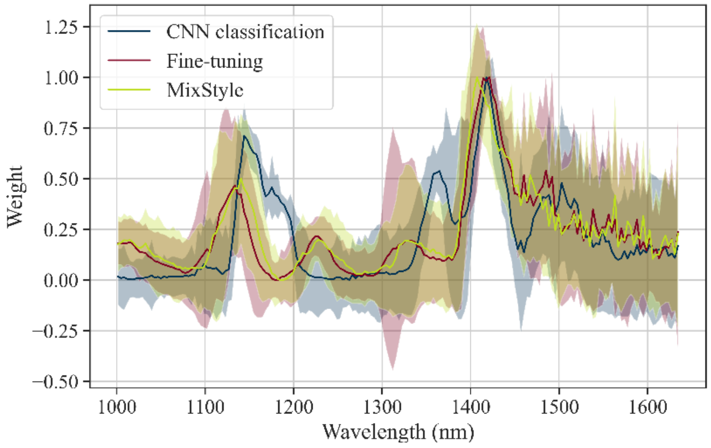

(C)

Figure. S4. Grad-CAM++ of each vigor gradient of CNN: Yongyou1540 transfer to Yongyou12: (A) the weights of not aged; (B) the weights of aging 96h; (C) the weights of aging 192h..

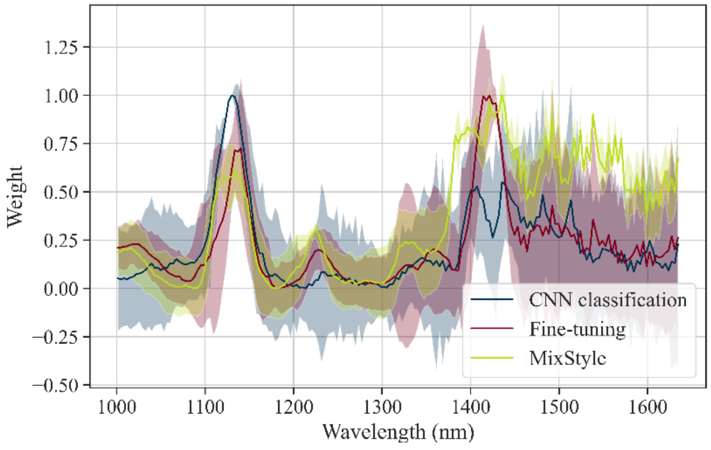

Weight
Wavelength (nm)
CNN classification
Fine-tuning
MixStyle

(A)

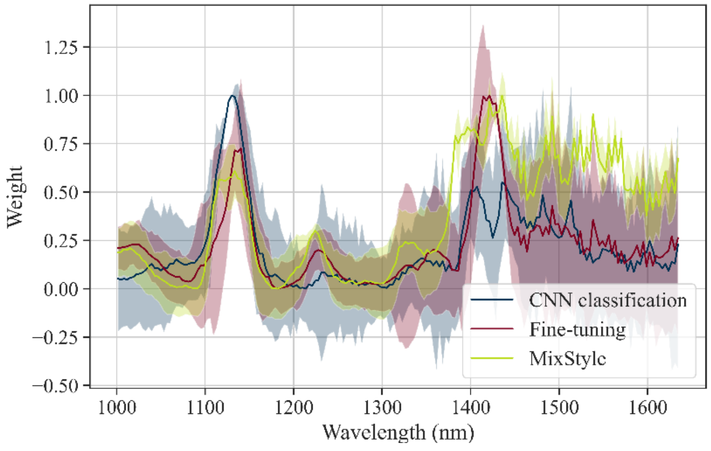

Weight
Wavelength (nm)
CNN classification
Fine-tuning
MixStyle

(B)

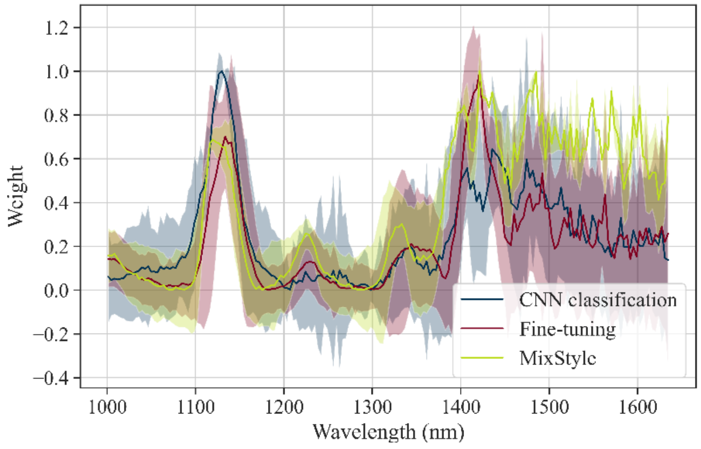

Weight
Wavelength (nm)
CNN classification
Fine-tuning
MixStyle

(C)

Figure. S5. Grad-CAM++ of each vigor gradient of CNN: Yongyou1540 transfer to Suxiangjing100: (A) the weights of not aged; (B) the weights of aging 96h; (C) the weights of aging 192h..

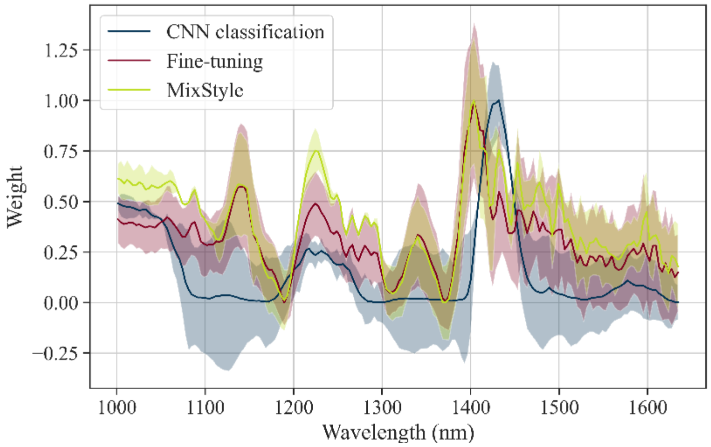

(A)

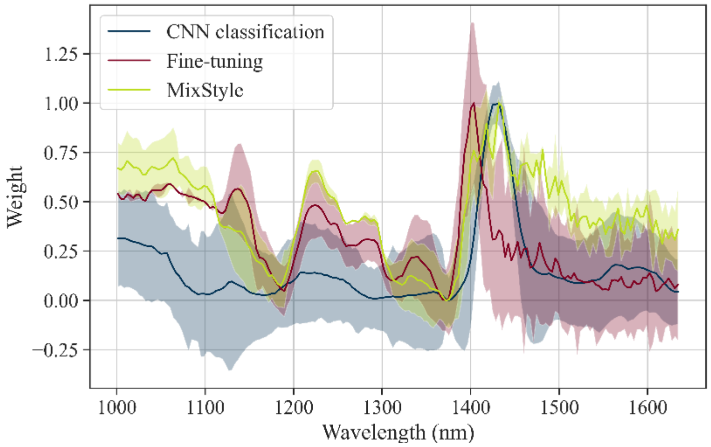

(B)

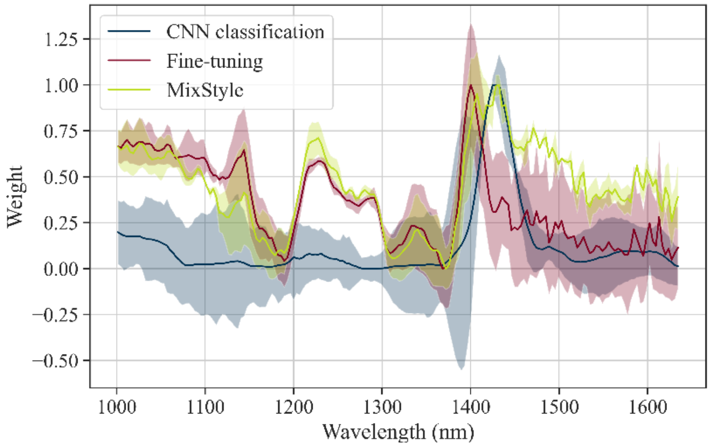

(C)

Figure. S6. Grad-CAM++ of each vigor gradient of CNN: Yongyou1540 transfer to Longjingyou1212: (A) the weights of not aged; (B) the weights of aging 96h; (C) the weights of aging 192h..

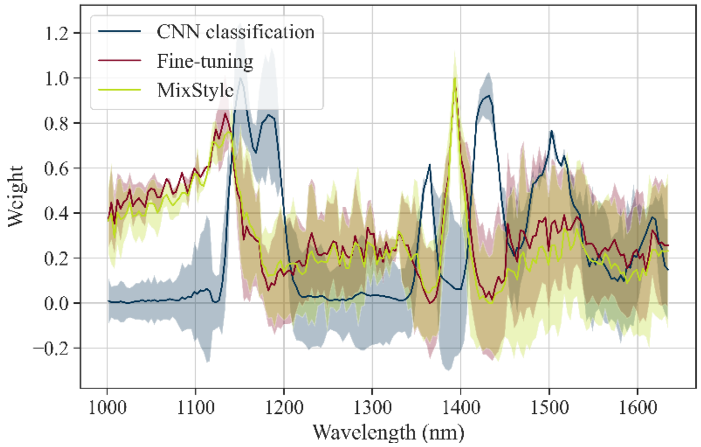

(A)

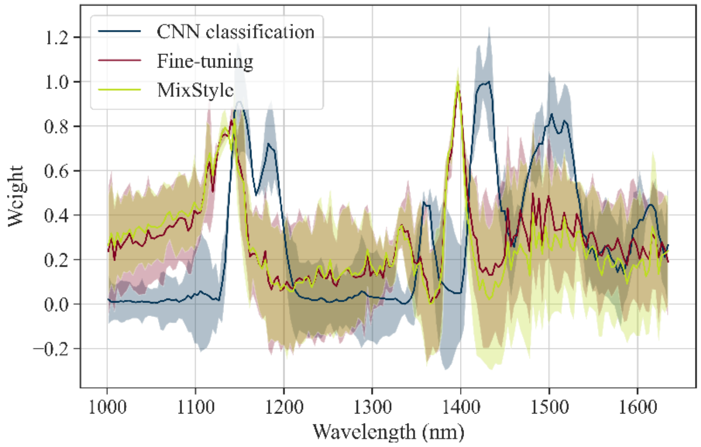

(B)

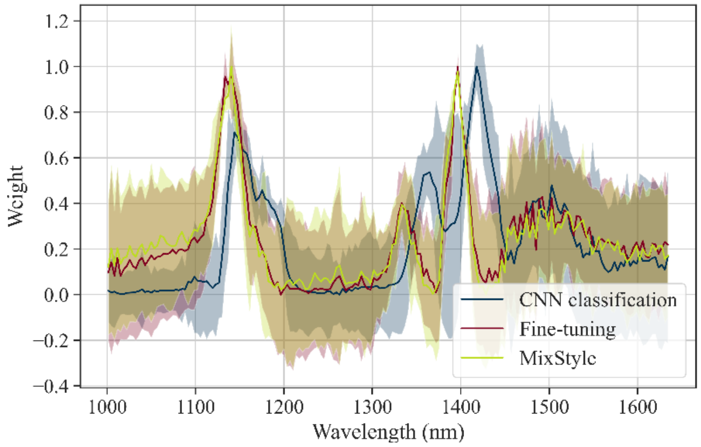

(C)

Figure. S7. Grad-CAM++ of each vigor gradient of CNN: Suxiangjing100 transfer to Yongyou12: (A) the weights of not aged; (B) the weights of aging 96h; (C) the weights of aging 192h..

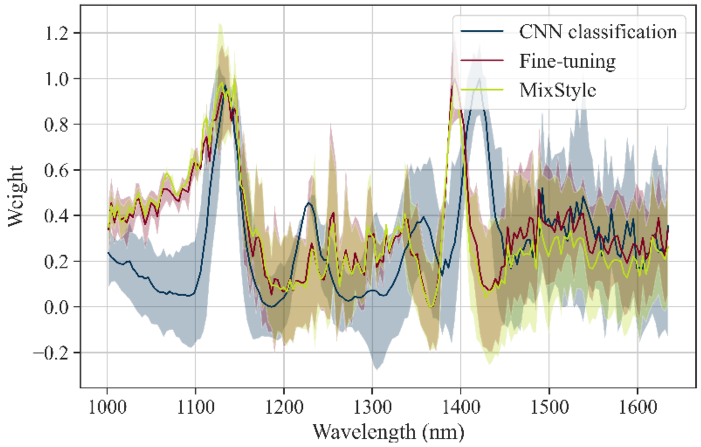
CNN classification
Fine-tuning
MixStyle
Weight
Wavelength (nm)

(A)

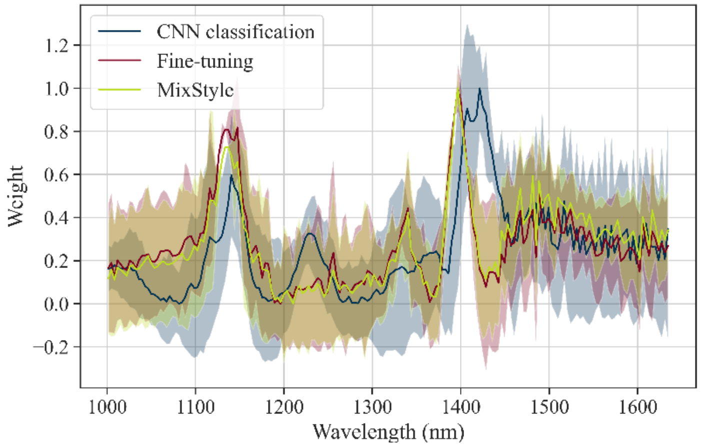
CNN classification
Fine-tuning
MixStyle
Weight
Wavelength (nm)

(B)

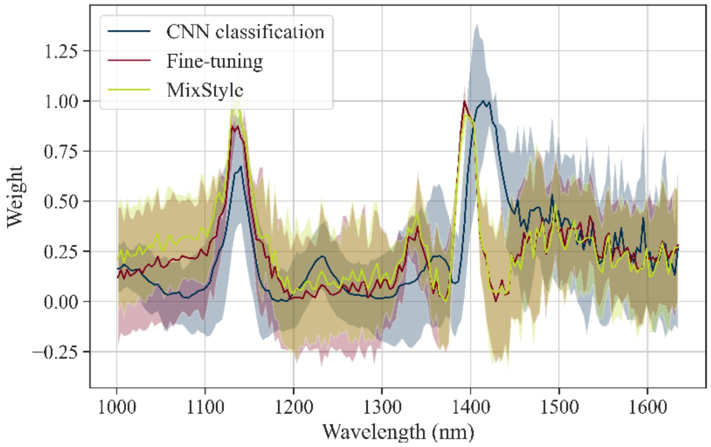
CNN classification
Fine-tuning
MixStyle
Weight
Wavelength (nm)

(C)

Figure. S8. Grad-CAM++ of each vigor gradient of CNN: Suxiangjing100 transfer to Yongyou1540: (A) the weights of not aged; (B) the weights of aging 96h; (C) the weights of aging 192h..

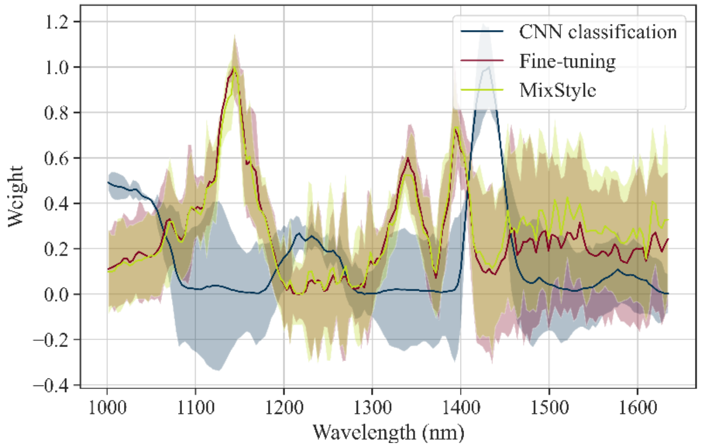

(A)

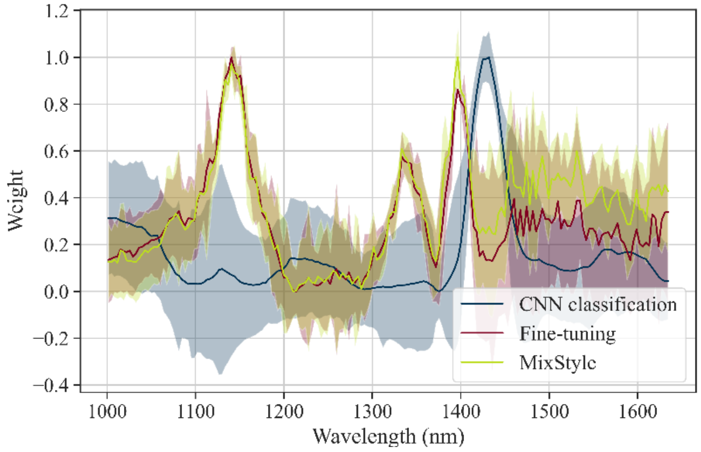

(B)

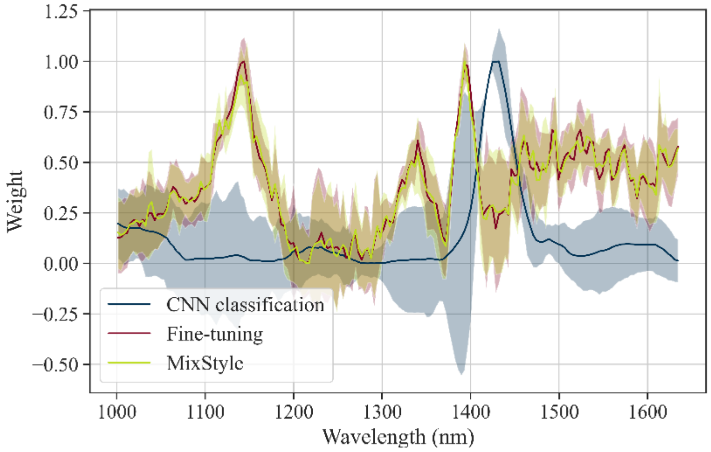

(C)

Figure. S9. Grad-CAM++ of each vigor gradient of CNN: Suxiangjing100 transfer to Longjingyou1212: (A) the weights of not aged; (B) the weights of aging 96h; (C) the weights of aging 192h..

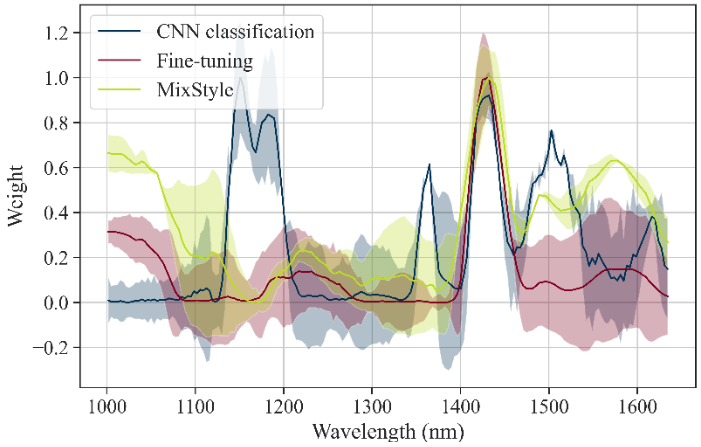

(A)

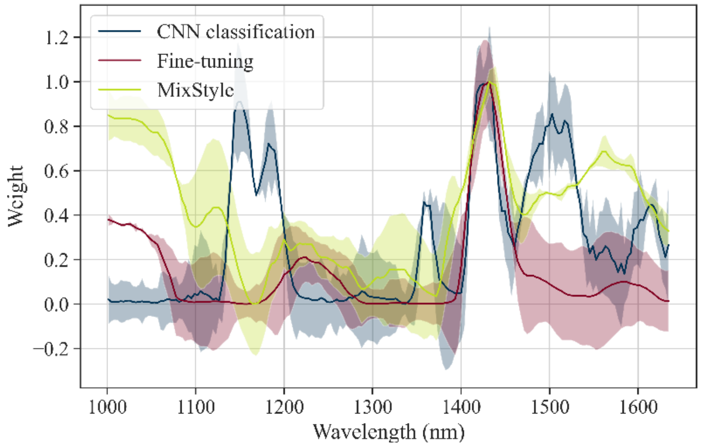

(B)

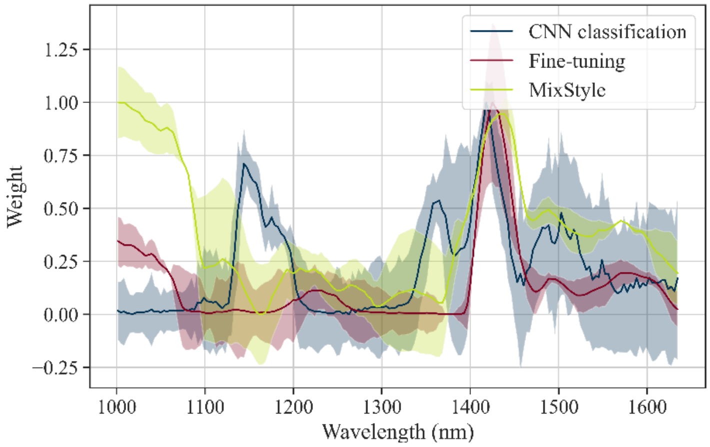

(C)

Figure. S10. Grad-CAM++ of each vigor gradient of CNN: Longjingyou1212 transfer to Yongyou12: (A) the weights of not aged; (B) the weights of aging 96h; (C) the weights of aging 192h..

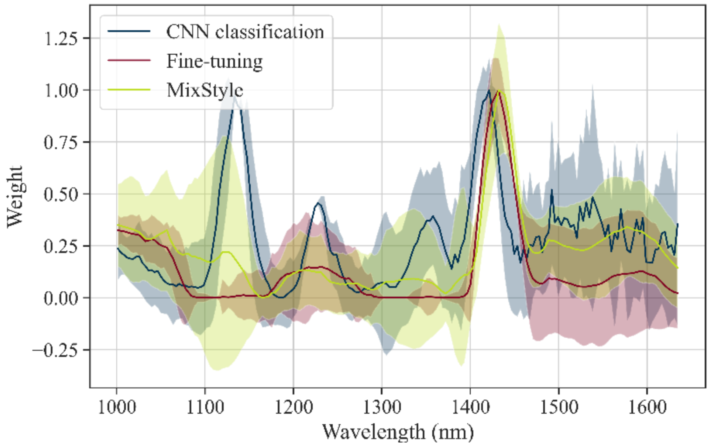
CNN classification
Fine-tuning
MixStyle
Weight
Wavelength (nm)
1.25
1.00
0.75
0.50
0.25
0.00
−0.25
1000
1100
1200
1300
1400
1500
1600

(A)

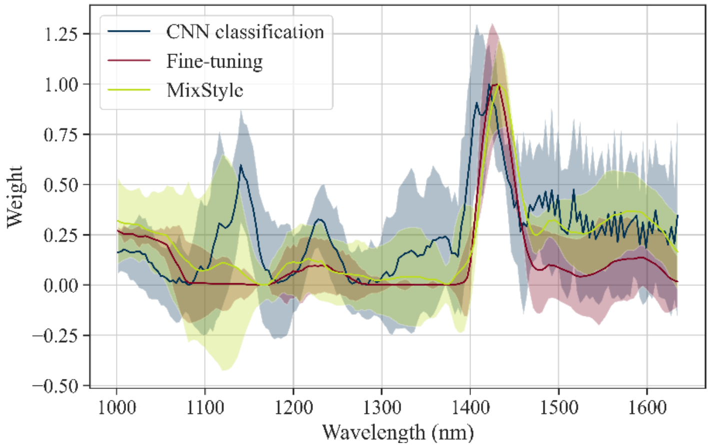
CNN classification
Fine-tuning
MixStyle
Weight
Wavelength (nm)
1.25
1.00
0.75
0.50
0.25
0.00
−0.25
−0.50
1000
1100
1200
1300
1400
1500
1600

(B)

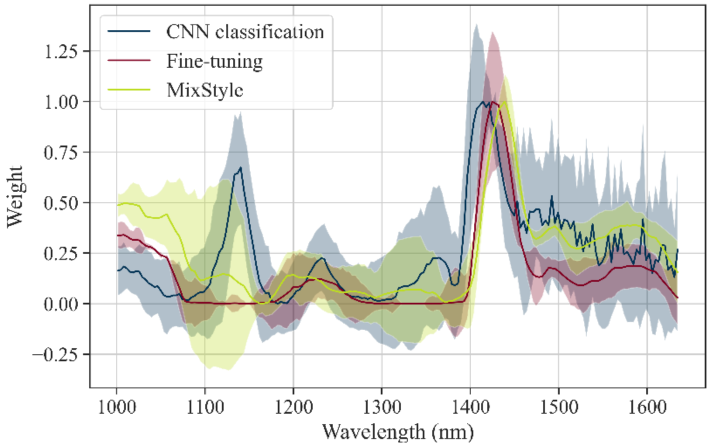
CNN classification
Fine-tuning
MixStyle
Weight
Wavelength (nm)
1.25
1.00
0.75
0.50
0.25
0.00
−0.25
1000
1100
1200
1300
1400
1500
1600

(C)

Figure. S11. Grad-CAM++ of each vigor gradient of CNN: Longjingyou1212 transfer to Yongyou1540: (A) the weights of not aged; (B) the weights of aging 96h; (C) the weights of aging 192h..

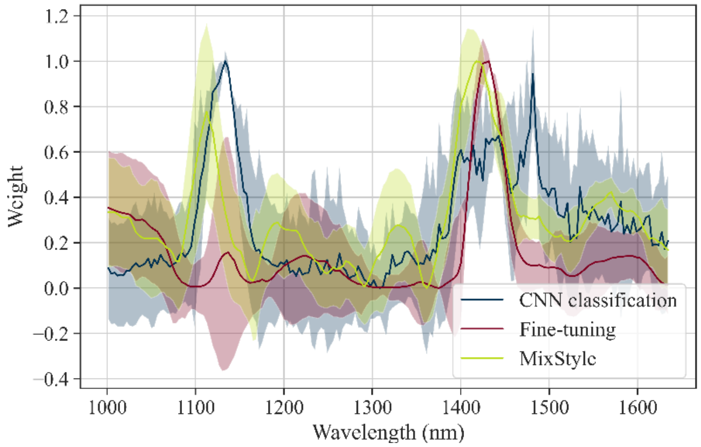

(A)

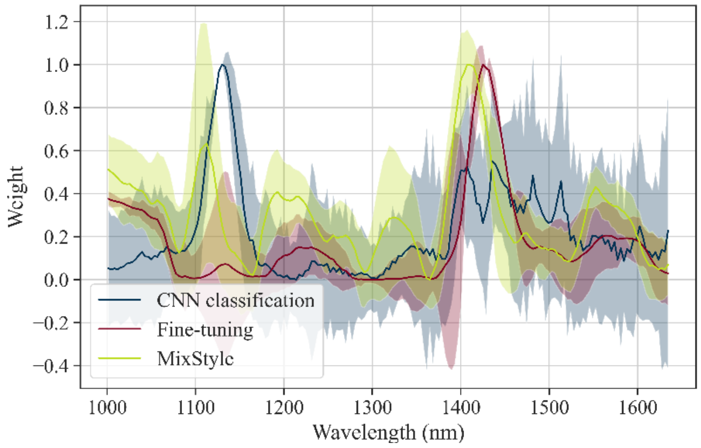

(B)

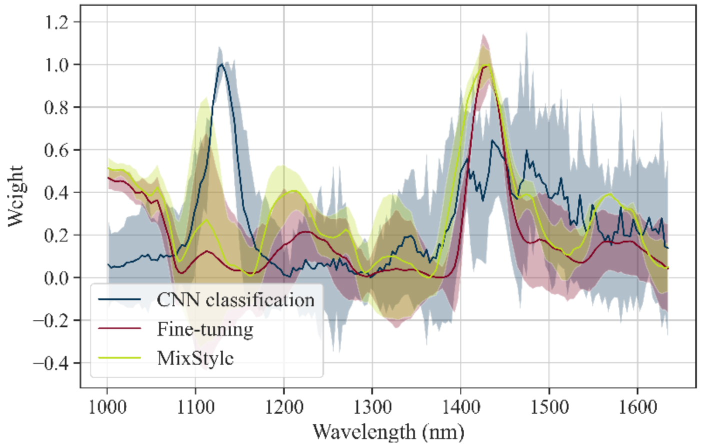

(C)

Figure. S12. Grad-CAM++ of each vigor gradient of CNN: Longjingyou1212 transfer to Suxiangjing100: (A) the weights of not aged; (B) the weights of aging 96h; (C) the weights of aging 192h..
